# PREVALENCE, IDENTIFICATION AND ANTIFUNGAL SUSCEPTIBILITY OF DERMATOPHYTES CAUSING TINEA CAPITIS IN A LOCALITY OF NORTH CENTRAL NIGERIA

**DOI:** 10.21010/ajid.v15i1.1

**Published:** 2020-12-14

**Authors:** Ekundayo Halimat Ayodele, Nwabuisi Charles**, Fadeyi Abayomi**

**Affiliations:** 1 Department of Medical Microbiology and Parasitology, University of Ilorin Teaching Hospital, Ilorin, Nigeria; 2 Department of Medical Microbiology and Parasitology, College of Health Sciences, University of Ilorin/ University of Ilorin Teaching Hospital, Nigeria

**Keywords:** *Tinea capitis*, prevalence, risk factors, dermatophytes, susceptibility pattern

## Abstract

**Background:**

*Tinea capitis* impacts negatively on the health of children, consequently affecting their education. Its prevalence is unknown in many African communities. *Tinea capitis* is faced with therapeutic challenges as resistance to all classes of antifungal agents continues to emerge. This study determined the prevalence, identified dermatophytes of *Tinea capitis* in Okelele community in North Central Nigeria; and evaluated the susceptibility of isolates to selected antifungal drugs.

**Materials and Methods:**

Three hundred and one pupils from seven primary schools in the locality who gave assent and those with parental consent were recruited into the study. Scalp scrapings and hairs were collected from participants and subjected to microscopy and culture. Isolates identified by colonial morphology and micromorphology were subjected to disk diffusion antifungal susceptibility testing.

**Results:**

Two hundred and twenty-eight of the participants had mycologically proven *Tinea capitis* giving a prevalence of 75.7%. The dermatophytes identified were *T. rubrum* (68.0%), *M. ferrugineum* (22.0%), *T. mentagrophytes* (8.0%) and *T. verrucosum* (2.0%). Resistance observed with these isolates was as low as 21.2% to as high as 100% while sensitivity ranged from 78.8% to 100%. Only large family size significantly influenced the occurrence of *T. capitis* among the risk factors.

**Conclusion:**

Prevalence of *Tinea capitis* from this study is high. *T. rubrum* being anthropophilic and the predominant dermatophyte identified corroborates large family size as an important risk factor. Antifungal resistance as a cause of therapeutic failure was demonstrated by some isolates in this study.

## Introduction

*Tinea capitis*, commonly called ring worm infection of the scalp, is predominantly seen among children, especially during the prepubescent age (Elewski, 2000). It affects the scalp hair follicles and surrounding skin causing significant hair loss (Nnoruka *et al.*, 2007). Scalp ring worm is highly contagious and frequently spread among family members and classmates (Ameneh, 2010). *Tinea capitis* has a global distribution but it is more prevalent in hot humid climates than temperate regions (Ameneh, 2010). It may occur sporadically or in epidemics. It is endemic in many developing countries making it a significant infectious dermatological disease (Nnoruka *et al.*, 2007). Males are more commonly affected than females, often with a frequency of about two to five times higher (Anosike *et al.*, 2005).

The causative agents of *T. capitis* vary from one geographical region to another, though largely due to *Microsporum* and *Trichophyton* species. The predominant dermatophyte causing *Tinea capitis* in a given geographical region may also change over time (Elewski, 2000), hence there is a need for constant surveillance to determine the epidemiological trends of the disease.

Despite advances in preventing and treating *Tinea capitis*, it remains a worldwide public health problem, especially among school children in economically underdeveloped countries (Ayanbimpe *et al.*, 2008). Acquired hair loss a common complication of the disease causes disfiguring and social stigma. In Nigeria, *T. capitis* is a major problem being endemic and also one of the most prevalent dermato-mycoses (Nweze, 2001). With the current global increase in antimicrobial resistance, therapy of *Tinea capitis* has also become a clinical challenge. The objective of this study therefore was to determine the prevalence and identify dermatophytes of *T. capitis* in Okelele community, a locality in North Central Nigeria and evaluate the susceptibility of isolates to selected antifungal drugs.

## Materials and Methods

This cross-sectional descriptive study was carried out in Okelele community, a locality in North Central Nigeria. Three hundred and one pupils aged 5-14 years attending the seven primary schools in the community were recruited via a multistage sampling technique. Ethical clearance was obtained from the Ethics and Research committee, University of Ilorin Teaching Hospital (U.I.T.H) vide a certificate UITH/CAT/189/14/239, and written informed consent from parent/guardian of children or assent of children older than seven years were obtained before recruitment.

Pupils with clinical evidence of *Tinea capitis* excluding those who have been on antifungal use within fourteen days prior to recruitment were selected. *Tinea capitis* was identified clinically by the presence of scaly scalp, alopecia or hair loss and pus filled sores (Ali *et al.*, 2007). A proforma designed for the study was used to obtain information on socio-demographic characteristics, clinical profile and risk factors.

Hairs and scalp scrapings were collected from each participant, into clean brown envelopes (Forbes *et al.*, 2007) while swab stick was used to obtain sample from pus filled sores (Winin *et al.*, 2006). All specimens were properly labeled to indicate the code for each subject, the type of specimen and date of collection. Specimens were then transported to the Mycology laboratory, Department of Microbiology, U.I.T.H., Ilorin and processed immediately.

A wet mount of each specimen was prepared in 20% potassium hydroxide (KOH). Each treated slide was examined under low(10x) and high (40x) objectives of a light microscope for the presence of spores and their distribution pattern in hairs (ectothrix, endothrix and favic type) and hyphae in scalp scrapings, crusts or pus. All specimens irrespective of microscopy results (whether positive or negative) were cultured on a pair of Mycosel agar plates (Monica, 2006). For each specimen one plate was incubated at room temperature and the other at between 35-37^o^C. The culture plates were examined every other day for evidence of growth for up to a maximum of four weeks thereafter cultures were considered negative for growth (Baron *et al.*, 2003). Isolates on culture were identified using colonial morphology and micromorphology using lactophenol cotton blue preparation. Macroscopic and microscopic features were compared to those contained in a practical mycology text (Ellis *et al.*, 2007). Further characterization of the isolates was done using the hair perforation and urease tests.

Isolates obtained were subjected to Agar Disk-diffusion antifungal susceptibility test (Rex *et al.*, 2006). The standardized inocula of the test organisms were prepared from fresh cultures made on chloramphenicol supplemented Sabouraud Dextrose Agar (SDA) slants (Barry *et al.*, 2000). On to each young culture of fungal isolate, 3mls of 0.9% normal saline was dispensed. With the aid of a sterile glass rod the dermatophyte isolates were gently scrapped to make a suspension. The organisms’ mixtures were filtered using whatmann filter paper No 1 to remove mycelia and hyphae.

The filtrate which contained the spores was then standardized using 0.5 Mac Farland turbidity standard that corresponds to a cell density of 1.5x10^8^ cfu/ml.Using sterile swab sticks SDA plates were then streaked evenly with the standardized inocula suspensions (Rex *et al.*, 2006). The antifungal discs namely Griseofulvin 25µg, Terbinafine 30µg, Itraconazole 8µg, Fluconazole 25µg by Oxoid, Ketoconazole 10µg, Miconazole 10µg and Clotrimazole 10µg from Mast Diagnostica were then applied to the surface of the inoculated agar plates. The inoculum of *T. mentagrophytes* ATCCMYA 4439 was similarly prepared and inoculated as control. All plates were incubated at room temperature for two to five days. Plates were examined daily for growth. After growth, the zones of inhibition for the test and control organisms were measured using a meter rule calibrated in millimeters. These measurements were compared with the CLSI standards.

Statistical analysis was performed using the Statistical Package for Social Sciences (SPSS) version 16 software. Descriptive analyses of variables were used to summarize data. The frequencies and mean (±sd) were generated for categorical and continuous variables respectively. Quantitative and qualitative demographic characteristics were summarized. Results were presented in tabular and pictorial forms. Categorical data were compared using Pearson’s chi square test. To determine the predictor(s) of the presence of *Tinea capitis*, binary logistic regression analysis was done. Odds ratio were determined with respective confidence intervals. A p-value < 0.05 was taken as statistically significant. Conclusions and recommendations were based on scientific evidence from the results.

## Results

A total of 301 pupils participated in this study and 228 had mycologically proven *Tinea capitis* giving a prevalence of 75.7% (Confidence Interval = 73.0-78.5).

Sixty-six (21.9%) out of the 301 participants were from Okelele A school, with the highest population of pupils; and the largest number 67 (22.3%) of the participants were from primary three class. Two hundred and fifty-nine (86.0%) of these children were males while 42 (14.0%) were females, giving a male to female ratio of 6.2: 1.0. The mean age of the pupils was 9.81±2.41. Nineteen (6.3%) of the pupils belonged to high socio-economic class (SEC) while majority 219 (72.8%) were from low SEC (Table 1).

**Table 1:** Socio-demographic characteristics of the Participants

Variable	Frequency (N=301)	Percentage (%)
**Primary Schools**		
Akerebiata	20	6.6
A.C.S. Sobi	22	7.3
Mogaji Are	27	9.1
Dada A	51	16.9
Dada B	52	17.3
Okelele B	63	20.9
Okelele A	66	21.9
**Primary School Classes**		
1	50	16.6
2	44	14.6
3	67	22.3
4	54	17.9
5	41	13.6
6	45	15.0
**Sex**		
Male	259	86.0
Female	42	14.0
**Age group (years)**		
5-9	132	43.9
10-14	169	56.1
Mean age + Sd	9.8 + 2.4	
**Socio-Economic Class**		
High	19	6.3
Low	282	93.7

Table 2 presents the prevalence of *Tinea capitis* according to age and sex. There was no significant difference in the infection rate between the two age groups (p=0.270) and across gender (p=0.275).

**Table 2:** Age and Sex prevalence of *Tinea capitis*

Age/sex	Number with Scalp lesions N=301	Number .positive(%)	*x^2^*	p-value
Age group (years)				
5-9	132	98(74.2)		
10-14	169	130(77.0)	11.089	0.270
Gender				
Male	259	199(76.8)		
Female	42	29(69.0)	1.193	0.275

**Figure 1:**
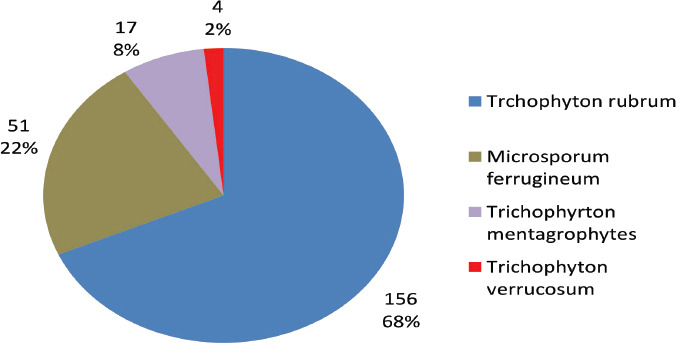
Fungal agents of *Tinea capitis*.

Figure 1 is a pie chart representing the aetiological agents of *Tinea capitis* among the school children. *Trichophyton rubrum* was the predominant isolate 156(68.9%), followed by *Microsporom ferrigineum* which accounted for 51(22.4%) of the isolates. *Trichophyton mentagrophytes* accounted for 17(7.4%) while *Trichophyton verrucosum* accounted for 4(1.8%) of the total isolates.

The antifungal susceptibility assay shows that 78.8% of the *T. rubrum* isolates were sensitive to clotrimazole and miconazole, 21.1% were resistant to both drugs, while all isolates (100.0%) of *T. rubrum* were resistant to ketoconazole, fluconazole, itraconazole ,griseofulvin, and terbinafine. *M. ferrugineum* and *T. mentagrophytes* were 100.0% sensitive to clotrimazole, miconazole and terbinafine. In addition, *T. mentagrophytes* showed 100.0% intermediate susceptibility to fluconazole. *T. verrucosum* was 100.0% resistant to all the antifungal drugs (Table 3).

**Table 3:** Susceptibility Pattern of the Fungal Agents of *Tinea Capitis*

Anti--fungal disc (µg)	*T. rubrum*			*Isolates/ (%)susceptibility M. ferrugineum*			*T. mentagrophytes*			*T. verrucosum*		
	**S**	**I**	**R**	**S**	** I**	**R**	**S**	**I**	**R**	**S**	**I**	**R**
GRS (25)	0(0.0)	0(0.0)	156(100.0)	0(0.0)	0(0.0)	51(100.0)	0(0.0)	0(0.0)	17(100.0)	0(0.0)	0(0.0)	4(100.0)
TER (30)	0(0.0)	0(0.0)	156(100.0)	51(100.0)	0(0.0)	0(0.0)	17(100.0)	0(0.0)	0(0.0)	0(0.0)	0(0.0)	4(100.0)
ICA (8)	0(0.0)	0(0.0)	156(100.0)	0(0.0)	0(0.0)	51(100.0)	0(0.0)	0(0.0)	17(100.0)	0(0.0)	0(0.0)	4(100.0)
FCA (25)	0(0.0)	0(0.0)	156(100.0)	0(0.0)	0(0.0)	51(100.0)	0(0.0)	17(100.0)	0(0.0)	0(0.0)	0(0.0)	4(100.0)
KCA (10)	0(0.0)	0(0.0)	156(100.0)	0(0.0)	0(0.0)	51(100.0)	0(0.0)	0(0.0)	17(100.0)	0(0.0)	0(0.0)	4(100.0)
MCL (10)	123(78.8)	0(0.0)	33(21.2)	51(100.0)	0(0.0)	0(0.0)	17(100.0)	0(0.0)	0(0.0)	0(0.0)	0(0.0)	4(100.0)
CTM (10)	123(78.8)	0(0.0)	33(21.2)	51(100.0)	0(0.0)	0(0.0)	17(100.0)	0(0.0)	0(0.0)	0(0.0)	0(0.0)	4(100.0)

ABBREVIATIONS: GRS – Griseofulvin; TER – Terbinafine; ICA – Itraconazole; FCA – Fluconazole; KCA – Ketoconazole; MCL – Miconazole; CTM – Clotrimazole; S –Sensitive; I – Intermediate Susceptibility, R—Resistant.

Table 4 shows that large family size had significant association with the occurrence of *T. capitis* (p< 0.05). Other risk factors were not significantly associated with its occurrence (p> 0.05). The odds of *Tinea capitis* occurring in children with large family size was two times higher compared to those with small family size (OR=2.025, 95% CI=1.185-3.461, p=0.010).

**Table 4:** Risk factors for Tinea capitis in the Children

Variable	Tinea capitis present N = 228 n (%)	Tinea capitis absent N = 73 n (%)	Total number sampled N = 301 n(%)	*x^2^*	OR	95% C I	p -value
**Socio-Economic Class**							
High	17 (7.5)	2 (2.7)	19 (6.3)	2.080	0.350	0.079-1.551	0.167
Low	211(92.5)	71 (97.3)	282 (93.7)				
**Family size							
< 5	74 (32.5)	36 (49.3)	110 (36.5)	6.778	2.025	1.185-3.461	*0.010
≥ 5 children	154(67.5)	37 (50.7)	191 (63.5)				
**Nutritional status**							
undernourished	127 (55.7)	38 (52.1)	165 (54.8)	0.297	0.863	0.509-1.464	0.586
Well-nourished	101 (44.3)	35 (47.9)	136 (45.2)				
**Similar scalp lesion in family members**							
Yes	152 (66.7)	43 (58.9)	195 (64.8)	1.460	0.717	0.417-1.232	0.228
No	76 (33.3)	30 (41.1)	106 (35.2)				
***Overcrowding							
Yes	125(54.8)	37 (50.7)	162 (53.8)	0.381	1.181	0.697-2.002	0.537
No	103(45.2)	36 (49.3)	139 (46.2)				
**Exposure to domesticanimals/pets**							
Yes	182(79.8)	58 (79.5)	240 (79.7)	0.005	0.977	0.508-1.878	0.945
No	46 (20.2)	15 (20.5)	61(20.3)				

KEY: *P < 0.05,

** ≥ 5 children per family is considered large family size (Adegbola, 2008),

*** ≥ 3 people living per room is taken as overcrowding (Park, 2005), OR = Odds ratio, C I= confidence interval.

## Discussion

The prevalence (75.7%) of *Tinea capitis* obtained, demonstrated a high infection rate among the school children. Several factors such as low standard of living, poor sanitary and hygienic conditions that are characteristics of rural communities especially in developing countries might have accounted for this. Lack of surveillance and control measures could also account for increased incidence of *T. capitis* over time. This prevalence was higher than the 31.2% reported for *Tinea capitis* by Ayanbimpe *et al*. (2005). The markedly lower prevalence obtained in previous studies (Menan *et al.*, 2002; Sidat *et al.*, 2007 ) represented prevalence of clinically suggestive *T. capitis* as opposed to prevalence of mycologically proven *Tinea capitis* among children with clinically suggestive disease reported in the present study. The variations in prevalence could also be due to the sampling methods used and could be a reflection of people’s habits, climatic conditions, standards of hygiene and levels of education all of which can influence predisposition to *Tinea capitis* (Fathi and Samarai, 2000). Adefemi *et al*. (2011) in a prevalence study among school children in Oke-oyi reported *T. capitis* as the predominant dermatophytosis where it was found to account for 76.1% of cases. This percentage, though comparable to the one obtained in the present study, represented the proportion of children with *T. capitis*. The prevalence of *Tinea capitis* was not influenced by age or sex. This probably indicates similar exposure to infection irrespective of age or sex.

The predominant causative agent of *Tinea capitis* among the children was *T. rubrum*. Others were *M. ferrugineum, T. mentagrophytes* and *T. verrucosum* in decreasing order. Out of these dermatophyte species *T. rubrum* and *M. ferrugineum* are anthropophilic, *T. verrucosum* is a zoophilic dermatophyte while *T. mentagrophytes* has both anthropophilic and zoophilic varieties. This finding shows a predominance of anthropophilic dermatophytes. The ecology of these dermatophytes indicates more commonly the transfer of infection from person to person and less commonly, from animals to humans. The affected children might be sharing personal care items such as combs, towels, hats and beddings which could serve as vehicles for the transfer of infective agents among them. Also, close interaction at home with infected family members and at school with infected playmates might have exposed them to infection with anthropophilic agents. Ayanbimpe *et al*. (2005) found *T. soudanese* as the predominant causative agent of *Tinea capitis* among other isolates. *T. mentagrophtes* was reported as the major isolate by Adefemi *et al*. (2011). In Turkey, Altindis *et al*. (2003) reported *T. violaceum* as the predominant isolate while in Ivory Coast, Menan *et al*. (2002) reported *T. soudanese* as the commonest etiological agent of *Tinea capitis*.

In Mozambique, *M. audouinii* was reported as the major isolate (Sidat *et al.*, 2007). These findings indicate variations over time, in the aetiological agents of *Tinea capitis* within the same geographical area and from one geographical area to the other as earlier reported (Ameh and Okolo, 2004; Sidat *et al.*, 2007 ).

Griseofulvin is the drug of choice for *Tinea capitis* and has been in use for several decades. All four dermatophyte species reported in this study were found to be resistant to griseofulvin. Repeated exposure of these fungal agents to griseofulvin and the long duration required for griseofulvin therapy with possible failure of compliance could account for the resistance. This observation was previously documented by Al-Refai (2007), thus suggesting possible reasons for treatment failure that may be experienced with the use of the drug. Similarly, all isolates were resistant to itraconazole and ketoconazole but *T. mentagrophytes* showed intermediate susceptibility to fluconazole. The relative safety and ease of delivery of the azoles has led to widespread use of these drugs both for prophylactic and therapeutic purposes. Repeated exposure to these drugs could result in secondary resistance (Rubio *et al.*, 2003). The intermediate susceptibility observed with fluconazole could mean that higher drug concentration would be needed to achieve therapeutic effect. Some studies (Pakshir *et al.*, 2007; Singh *et al.*, 2007) however, have documented fluconazole to have less activity against dermatophytes. With the exception of *T. verrucosum*, the isolates were sensitive to miconazole and clotimazole. Mukherje *et al*. (2003) documented primary resistance of *T. rubrum* to terbinafine and this might suggest why all the *T. rubrum* isolates in this study could have intrinsic resistance to terbinafine. Although *T. mentagrophytes* and *M. ferrugineum* were sensitive to terbinafine, terbinafine is not commonly used for the treatment of *Tinea capitis*.

*Tinea capitis*, like any contagious disease, has associated risk factors. This study found a significant association between family size and *Tinea capitis*. Children from large families were twice as likely to have *Tinea capitis* compared to those with small families. Large family size with limited income for family upkeep encourages the sharing of personal care items. These items could act as vehicles facilitating the transfer of fungal spores from one infected child to the other which could lead to spread of the disease. With inadequate living space, large family size could result in overcrowding. This may encourage close interaction between infected and non-infected family members leading to transmission of infection, although this study did not find statistically significant association between overcrowding and *Tinea capitis*. Other risk factors such as socio-economic class, malnutrition, presence of scalp lesions in family members and exposure to domestic animals and pets were not significantly associated with *Tinea capitis*. These findings are in contrast with reports from previous studies (Nweze, 2001; Ameh and Okolo, 2004;Adefemi *et al.*, 2011) where these risk factors were found to be associated with *Tinea capitis*.

## Conclusion

Prevalence of *Tinea capitis* among school children in the locality is high. Infection was caused mostly by anthropophilic dermatophytes and large family size was the main predisposing factor for *Tinea capitis*. These findings stress the importance of good personal hygiene and improved standard of living as measures necessary in the prevention of *T. capitis*. Health education in the schools on the mode of transmission, with emphasis on the identified risk factors and ways of preventing *Tinea capitis* is recommended and should form part of the school health programme in all primary schools in the community.

### Authors’ Contributions

Ekundayo Halimat Ayodele conceptualized the work and collected the data. Fadeyi Abayomi carried out data analysis. Nwabuisi Charles gave outline on manuscript writing and suggested the journal. Ekundayo Halimat Ayodele prepared the manuscript. All authors read and agreed on the manuscript.

**List of Abbreviations:**
(ATCC): America Type Culture Collection
(CLSI): Clinical and Laboratory Standards Institute
(KOH): Potassium hydroxide
(SDA): Sabouraud Dextrose Agar
(SEC): Socio-economic Class
(SPSS): Statistical Packages for Social Sciences
(UITH): University of Ilorin Teaching Hospital
